# Differentiation of embryonic stem cells into a putative hair cell-progenitor cells via co-culture with HEI-OC1 cells

**DOI:** 10.1038/s41598-021-93049-3

**Published:** 2021-07-06

**Authors:** Nathaniel T. Carpena, So-Young Chang, Celine D. G. Abueva, Jae Yun Jung, Min Young Lee

**Affiliations:** 1 Department of Otolaryngology-Head and Neck Surgery, College of Medicine, Dankook University, 119 Dandae-ro, Cheonan, 31116 Republic of Korea; 2 Medical Laser Research Center, Dankook University, Cheonan, Republic of Korea; 3 Beckman Laser Institute Korea, Dankook University, 119 Dandae-ro, Cheonan, 31116 Republic of Korea

**Keywords:** Cell biology, Neuroscience, Stem cells, Neurology

## Abstract

Several studies have shown how different cell lines can influence the differentiation of stem cells through co-culture systems. The House Ear Institute-Organ of Corti 1 (HEI-OC1) is considered an important cell line for in vitro auditory research. However, it is unknown if HEI-OC1 cells can promote the differentiation of embryonic stem cells (ESCs). In this study, we investigated whether co-culture of ESCs with HEI-OC1 cells promotes differentiation. To this end, we developed a co-culture system of mouse ESCs with HEI-OC1 cells. Dissociated or embryonic bodies (EBs) of ESCs were introduced to a conditioned and inactivated confluent layer of HEI-OC1 cells for 14 days. The dissociated ESCs coalesced into an EB-like form that was smaller than the co-cultured EBs. Contact co-culture generated cells expressing several otic progenitor markers as well as hair cell specific markers. ESCs and EBs were also cultured in non-contact setup but using conditioned medium from HEI-OC1 cells, indicating that soluble factors alone could have a similar effect. The ESCs did not form into aggregates but were still Myo7a-positive, while the EBs degenerated. However, in the fully differentiated EBs, evidence to prove mature differentiation of inner ear hair cell was still rudimentary. Nevertheless, these results suggest that cellular interactions between ESCs and HEI-OC1 cells may both stimulate ESC differentiation.

## Introduction

Sensorineural hearing loss is developed due to the loss of cochlear hair cells, which are responsible for converting mechanical sound vibrations into electrical signals to the brain via mechano-electrical transduction^1^. Unlike other epithelial tissues of the human body, the sensory epithelium of the cochlea is composed of cochlear hair cells and various other cell types that cannot regenerate^2^. This unfortunate fate of cochlear hair cells leads to abrupt or progressive hearing loss over time in mammals, which cannot be rivaled by any medical or surgical treatment.

The mammalian hearing organ has an organized architecture, in which lines of inner and outer hair cells are surrounded by a variety of supporting cells on the basilar membrane of the organ of Corti (OC). Conventional monoculture systems are unable to recapitulate the physiological and biological interactions in vivo. Applying stem cells into the co-culture system could be important for the creation of complex organs^3^ such as the OC. Several studies have shown that stem cells are able to differentiate into various cell types when co-cultured with specific cell types. Co-culture systems have been used in tissue engineering to study cell-to-cell interactions between populations as well as to establish new interactions with other cell populations in hopes of influencing the maintenance or transformation of certain populations^4^.

Direct co-culture is the cultivation of stem cells in direct physical contact with another cell type closely related to the target organ but without exogenous stimulators^5,6^. Meanwhile, indirect co-culture uses soluble factors released by the other cell type to direct the differentiation of stem cells without cell-to-cell contact^7–9^. Aside from organotypic cell cultures of the inner ear, House Ear Institute-Organ of Corti 1 (HEI-OC1) cells bear the characteristics of cells of the sensory epithelium and are considered one of the most important cell lines available for in vitro auditory research. HEI-OC1 cells have been the staple for investigating molecular pathways and pharmacological treatments related to hearing loss^10–12^. However, it is unknown if HEI-OC1 cells can promote the differentiation of embryonic stem cells (ESCs).

In this study, we conditioned HEI-OC1 cells to survive in the stem cell culture environment and developed both direct and indirect stem cell co-culture systems. We determined that both co-culture systems generated myosin VIIa (Myo7a)-positive cells and compared otic differentiation gene expression levels between a co-culture system and a conventional otic differentiation protocol.

## Results

### Conditioned HEI-OC1 cells maintain auditory cell characteristics

**Figure 1:**
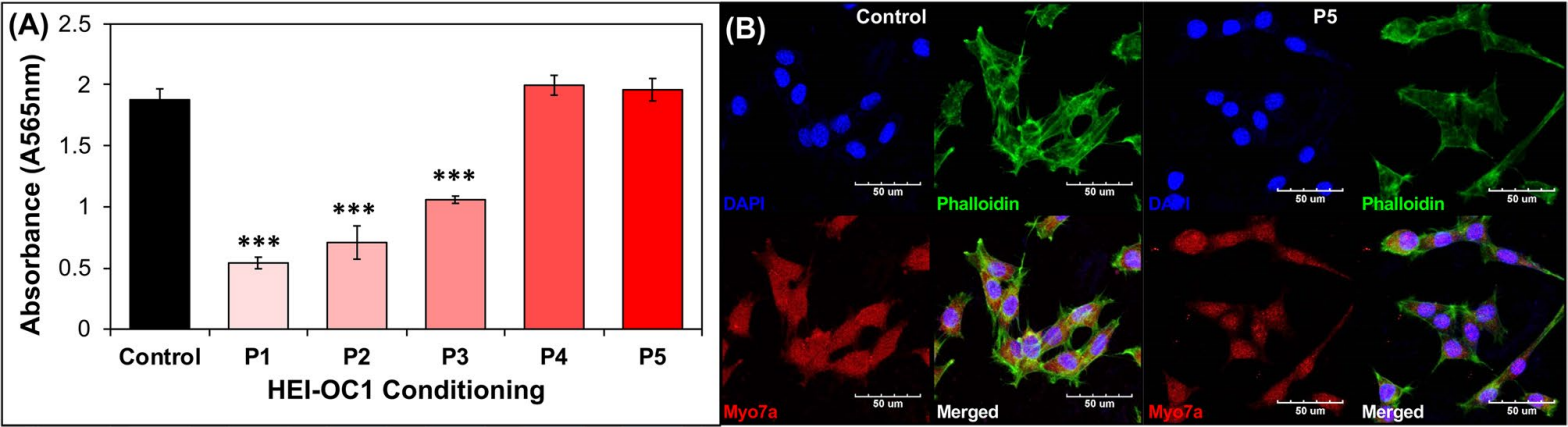
HEI-OC1 cell conditioning for co-culture. HEI-OC1 cells were cultured in stem cell media at 37 °C and passaged five times prior to co-culture. (**A**) MTT assay showed a decrease in cell proliferation at the start of conditioning. Proliferation of conditioned HEI-OC1 cells normalized with the control after the fourth passage. (**B**) Confocal images of HEI-OC1 cells before (Control) and after conditioning (P5); expression of f-actin and Myo7a was observed at both time points. Scale bars represent 50 µm.

To maintain the viability of HEI-OC1 cells, unique culture conditions are required. The temperature and carbon dioxide concentration for HEI-OC1 cell culture is different from the culture conditions of stem cells. To culture both HEI-OC1 cells and stem cells together, modification of the culture conditions should be considered. In the present study, we pre-conditioned HEI-OC1 cells to survive in stem cell culture conditions as described in the Materials and Methods. Initial passage of HEI-OC1 cell culture at 37 °C and 5% CO_2_ cultured in ESCs medium had significantly low proliferative capacity relative to unconditioned cells kept at its typical medium (DMEM with 10% FBS) and incubated at 33 °C and 10% CO_2_. However, the proliferative capacity of each surviving colony from successive passages recovered after only four passages (Fig. 1A). After five passages of conditioning, both cell proliferation and morphology were comparable to the unconditioned group (Fig. 1B) with a 98 ± % cell proliferation cultured in ESC media incubated at 37 °C and 5% CO_2_. Conditioned HEI-OC1 cells retained their morphology as observed by f-actin staining and otic hair cell specific marker Myo7a. These successfully conditioned HEI-OC1 cells were then used for co-culturing with mESCs in subsequent experiments.

### Contact co-culture generates Myo7a- and Sox2-positive cellular structures from both dissociated mESCs and EBs

**Figure 2:**
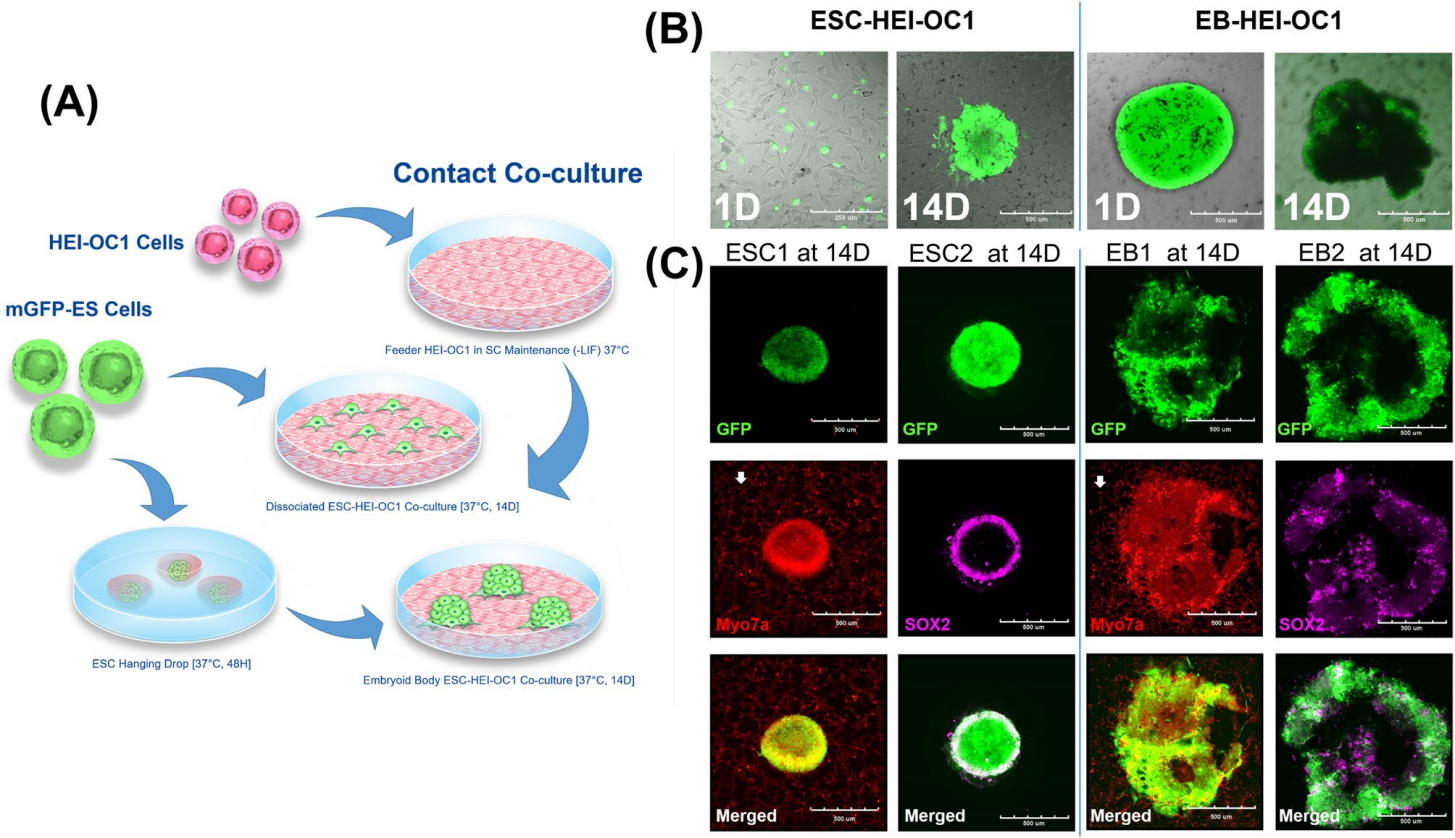
Contact co-culture of dissociated embryonic stem cells (ESC)s and embryoid bodies (EB)s with HEI-OC1 cells induced differentiation into otic cells. (**A**) Methodology of contact co-culture is shown. HEI-OC1 cells were preconditioned as described in the “Materials and methods” and co-cultured with both dissociated ESCs and EBs generated by hanging drop for 14 days. (**B**) Representative image of two different co-cultures. Dissociated ESCs merged and formed aggregates at 14 days. Protruding structures at the periphery of EBs were observed after 14 days. (**C**) Epifluorescence analysis showing expression of otic marker Myo7a and neural marker SOX2. Myo7a and SOX2 were both expressed in aggregates formed by dissociated ESC culture and EBs. Overlap expression of GFP (from ESCs), Myo7a, and SOX2 were observed in both groups. Co-cultured HEI-OC1 cells were only stained by Myo7a (white arrow).

In the case of contact co-culture, conditioned HEI-OC1 cells were incubated with GFP-positive dissociated mESCs and EBs, which were generated by mESCs using the hanging drop technique (see “Materials and methods”) in the stem cell media for 14 days (Fig. 2A). On day 1 of co-culture, morphologies of both dissociated mESCs and EBs were similar to the non-contact co-culture. After 14 days, dissociated mESCs in contact co-culture formed cellular aggregates and generated EB-like structures (Fig. 2B). On the other hand, EBs after 14 days of contact co-culture exhibited changes in morphology, showing several protruding structures from the surface, which resembled generated otic organoids from a prior report^13^. Each cellular structure generated from contact co-culture of dissociated mESCs or EBs expressed GFP, and Myo7a and Sox2 markers (Fig. 2C). In the case of the aggregates generated from contact co-culture of dissociated mESCs, expression of Myo7a and Sox2 was highly observed in the peripheral part of the structure, which is in contact with co-cultured HEI-OC1 cells (Myo7a-positive dissociated cells; arrow). In contrast, structures from contact co-culture of EBs, showed scattered Myo7a- and Sox2-positive areas as compared to peripheral expression observed in structures generated from dissociated mESCs (Fig. 2C). These results suggest that co-culture of conditioned HEI-OC1 cells with either dissociated mESCs or EBs results in differentiation to otic cell-like structures or cells.

### EBs from contact co-culture showed markers of hair cell progenitors but failed to generate hair cell-like structures

**Figure 3:**
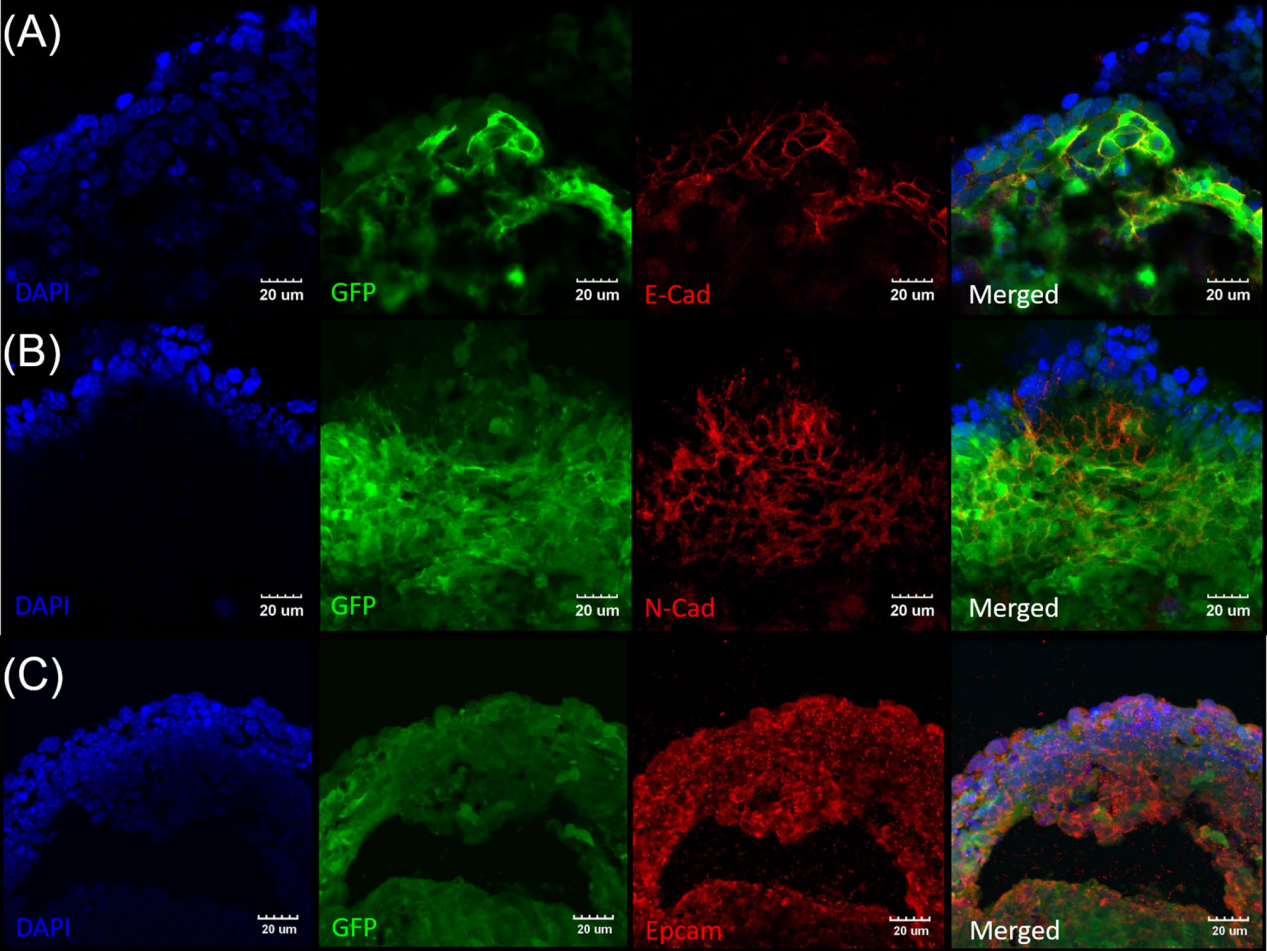
Contact co-culture of ESC-EBs with HEI-OC1 cells generated prosensory epithelia. EBs from contact co-culture were isolated after 14 days and sectioned and stained for the presence of ectodermal differentiation. Representative high magnification images of (**A**) EB showing sparse expression of GFP^+^/E-Cad^+^ cells. (**B**) Patches of GFP^+^/N-Cad^+^ expression within the co-cultured EBs. (**C**) GFP^+^/Epcam^+^ expression within the EBs mark the prosensory epithelia. Images are representative of EBs isolated from at least three separate experiments. Scale bars are 20 µm.

The differentiated EBs formed via contact co-culture for 14 days were further analyzed for staining of markers specific to otic hair cells. EBs generated patches of epithelial cadherin positive (E-cad^+^, Fig. 3A) cells as well as neural cadherin positive (N-cad^+^, Fig. 3B) cells showing possible ectodermal morphology. EBs were also positive for the epithelial cell adhesion molecule (Epcam^+^) marking the prosensory epithelia Fig. 3C).

**Figure 4:**
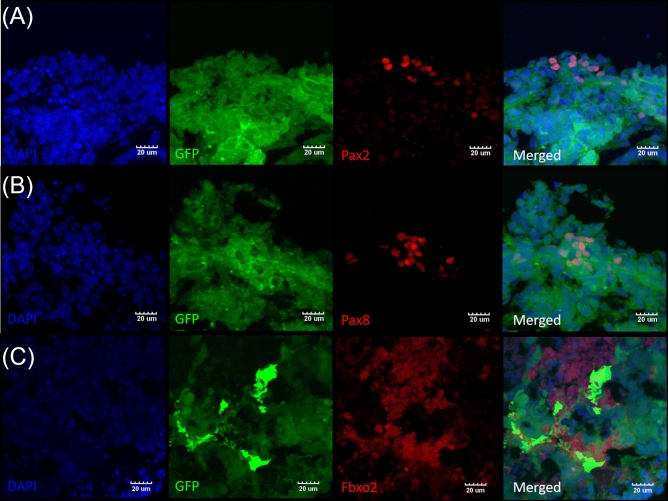
ESC-EBs differentiated into otic progenitor cells via contact co-culture with HEI-OC1 cells. Representative high magnification images of (**A**) Pax2^+^, (**B**) Pax8^+^ and (**C**) Fbxo2 cells in GFP^+^ EBs indicate otic progenitor's presence. Images are representative of EBs isolated from at least three separate experiments. Scale bars are 20 µm.

There are also an occasional presence of patches paired box protein 2 and 8 positive (Pax2^+^ and Pax8^+^, Fig. 4A,B) as well as the ubiquitin ligase Fbxo2 (Fig. 4C) indicating the development of the otic progenitors. Within these possible sensory epithelia, co-cultured EBs also generated cells that shows multiple hair cell markers. We have failed to observe well organized expression of the further mature otic markers such as Brn3c or Anxa4. Neither characteristic structures of otic hair cells such as stereocilia was not identified.

### Non-contact co-culture generates Myo7a-positive cells from dissociated mESCs but did not form aggregates

**Figure 5:**
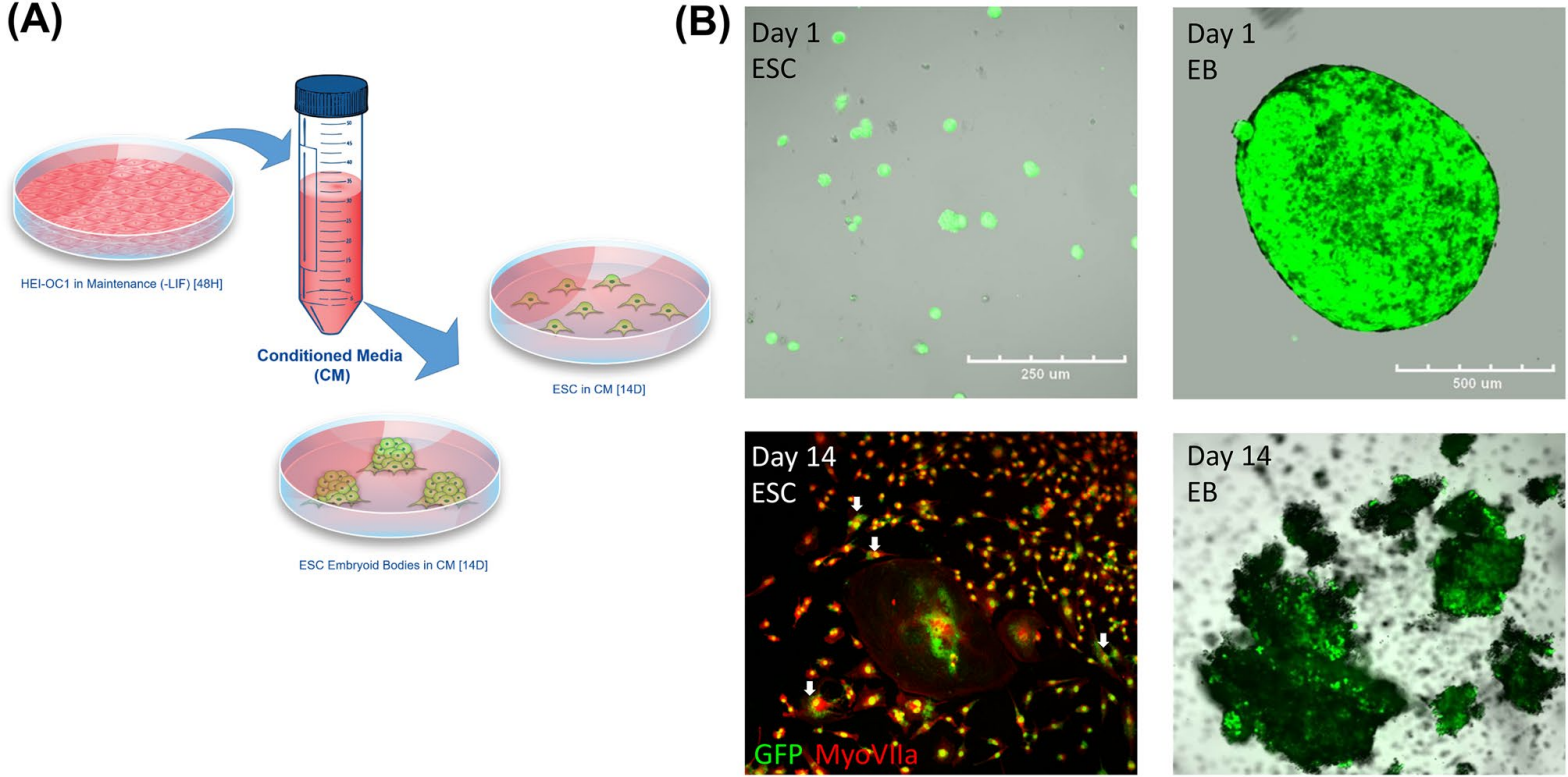
Non-contact co-culture of dissociated ESCs with HEI-OC1 cells induced differentiation into otic cells. (**A**) Methodology of non-contact co-culture is shown. After inoculating pre-conditioned HEI-OC1 cells in culture media for 48 h, soup of the culture was acquired and added to dissociated ESCs and EBs generated by hanging drop. Both dissociated ESCs and EBs were cultured with CM for 14 days. (**B**) Upper row is a light microscopy image of dissociated ESCs and EBs at day 1 of culture with CM, showing GFP expression of ESCs. Bottom row is a microscopy image at day 14 in each group. Dissociated ESCs show Myo7a expression, and co-expression of Myo7a and GFP is depicted by white arrows. By contrast, EBs failed to maintain the structure and broke after 14 days. Scale bars for each column are located in the top row.

After incubating HEI-OC1 cells in the stem cell media for 48 h, soup of the culture without cells (conditioned media, CM) was taken and provided to dissociated mESCs and EBs. Both dissociated mESCs and EBs were incubated for 14 days in CM without any additional procedures or applications (Fig. 5A). On day 1 of non-contact co-culture, dissociated mESCs showed normal morphology and colony formation. EBs also showed normal morphology without any structural change at day 1 (Fig. 5B, upper row). After 14 days of non-contact co-culture, dissociated mESCs did not form aggregate structures unlike in contact co-culture but showed similar morphologies to HEI-OC1 cells. Co-expression of Myo7a and GFP was also observed in many cells, suggesting Myo7a-positive cells were derived from mESCs and confirming otic differentiation (Fig. 5B, lower row). In contrast, after 14 days in CM co-culture, EBs failed to maintain their structure (Fig. 5B, lower row).

### EBs co-cultured with HEI-OC1 cells express otic hair cell related markers

**Figure 6:**
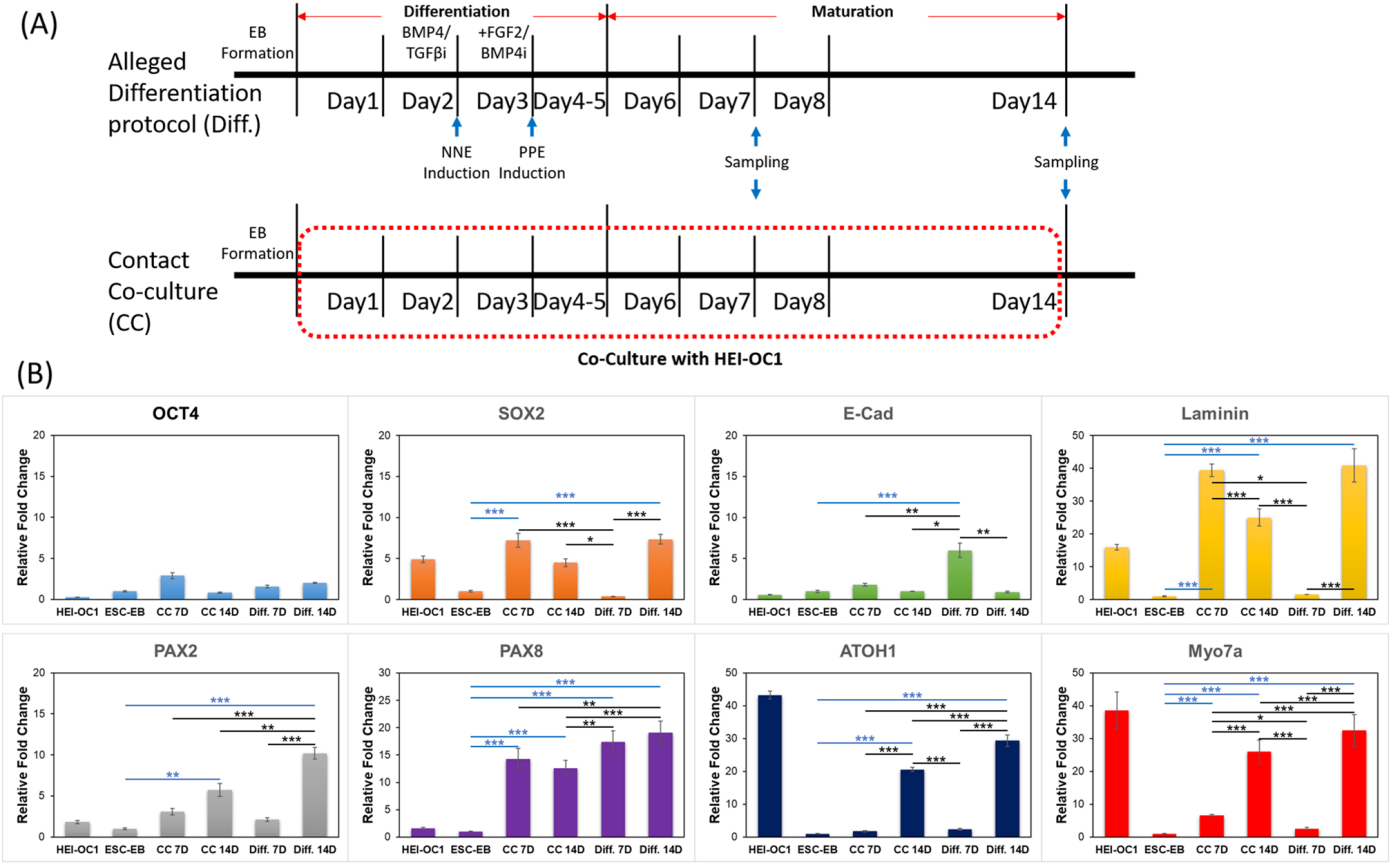
RT-PCR analysis of co-cultured EBs. (**A**) Two different processes of differentiation. The top image shows the differentiation (Diff) protocol for otic organoids and, the bottom image shows the protocol for contact co-culture (CC). (**B**) In both differentiation protocols, EBs are harvested at 7 and 14 days after initiation of differentiation. (**B**) Oct4 was not detected in HEI-OC1 cells. SOX2 was detected in all stem cells and significantly increased compared to non-differentiated ESCs in CC on day 7 and 14 and in Diff at day 14. E-Cad was detected in all groups and significantly increased compared to non-differentiated ESCs only in Diff at day 7. A significant increase in Laminin was observed compared to non-differentiated ESCs in CC at day 14 and in Diff at day 7 and 14. Significant increases in PAX2, ATOH1, and Myo7a compared to non-differentiated ESCs was observed in CC and Diff at day 14. HEI-OC1 cells showed significantly increased expression of ATOH1 and Myo7a compared to non-differentiated ESCs. Statistical analysis determined by one-way ANOVA with Dunnett test (GraphPad Software ver. 9.0, Inc., La Jolla, CA, USA). *p < 0.05, **p < 0.01, and ***p < 0.001. Differences compared to ESCs-EB are shown in blue asterisks.

To evaluate the characteristics of EBs with HEI-OC1 cells by contact co-culture, which appeared to be relatively heterogenous. RT-PCR analysis was also conducted in order to quantitatively analyze the expression of otic hair cell related genes. As a comparison, otic organoids generated from EBs (mESCs) by an established protocol^14^ were used for conventional differentiation. The differentiation protocol for control otic organoids and procedures for contact co-culture is shown in Fig. 6A. Specimens were harvested at early (7 days) and late (14 days) stages of the differentiation process. Relative fold changes were compared to undifferentiated mESCs. Multiple genes related to differentiation of the otic lineage are shown in Fig. 6B. E-Cad expression was only robust in the early stage and SOX2 expression was observed only in the late stage of conventional differentiation. On the other hand, E-Cad expression also elevated on the early stage of the co-culture although not statistically significant and SOX2 was observed in both the early and late stages. These results suggest that differentiation using the conventional method and co-culture are not identical. Laminin and PAX8 expressions were observed with both differentiation processes. PAX2, ATOH1, and Myo7a expressions were observed with both differentiation processes at the late stage. Detailed information of the statistical analyses is provided in Table 1. These results indicate the successful differentiation of otic structures (organoids) by conditioned HEI-IOC1 cell co-culture without additional modifications.

**Table 1:** Statistical analysis of levels of mRNA expression for HC differentiation-related genes using RT-qPCR.

Target genes	2 Way ANOVA (*p* value)	ESC-EB vs CC 7D	ESC-EB vs CC 14D	ESC-EB vs Diff. 7D	ESC-EB vs Diff. 14D	CC 7D vs CC 14D	CC 7D vs Diff. 7D	CC 7D vs Diff. 14D	CC 14D vs Diff. 7D	CC 14D vs Diff. 14D	Diff. 7D vs Diff. 14D
OCT4	p > 0.05 (ns)	ns	ns	ns	ns	ns	ns	ns	ns	ns	ns
SOX2	p > 0.05 (ns) p < 0.001 (***) p < 0.05 (*)	***	ns	ns	***	ns	***	ns	*	ns	***
E-Cad	p > 0.05 (ns) p < 0.05 (*) p < 0.01 (**)	ns	ns	**	ns	ns	*	ns	**	ns	**
Laminin	p > 0.05 (ns) p < 0.001 (***)	***	***	ns	***	***	***	ns	***	***	***
PAX2	p > 0.05 (ns) p < 0.01 (**) p < 0.001 (***)	ns	**	ns	***	ns	ns	***	ns	**	***
PAX8	p > 0.05 (ns) p < 0.01 (**) p < 0.001 (***)	***	***	***	***	ns	ns	**	**	***	ns
M7A	p > 0.05 (ns) p < 0.05 (*) p < 0.001 (***)	***	***	ns	***	***	*	***	***	***	***
ATOH1	p > 0.05 (ns) p < 0.001 (***)	ns	***	ns	***	***	ns	***	***	***	***

## Discussion

Since the discovery of the pluripotency of stem cells, differentiation strategies have been at the forefront of tissue engineering and regeneration. Cell differentiation techniques of pluripotent cells such as ESCs into cochlear hair cells have been investigated to restore hearing. The most straightforward and common way to differentiate ESCs is into hair cells is via well-timed and gradual addition of bulk molecules. Conventional differentiation protocols for the generation of inner ear hair cells from stem cells in vitro are multi-step and require the well-timed addition of several exogenous bioactive molecules, such as BMP4, transforming growth factor β inhibitor, FGF2, and Wnt^13–15^. We posited that co-culture using HEI-OC1 cells could induce otic differentiation of EBs. Our study was able to produce premature hair cell-progenitor cells through contact co-culture of EBs with conditioned HEI-OC1 cells which provided the necessary factors for differentiation.

HEI-OC1 cells are derived from the inner ear of the transgenic mouse Immortomouse™ in which the immortalizing gene could be activated depending on a difference in temperature^16^. With a permissive condition at 33 °C and 10% CO_2_, the immortalizing gene is activated, allowing cells to proliferate. Without gene activation at 37–39 °C and 5% CO_2_ (non-permissive condition), these cells spontaneously differentiate and eventually undergo apoptosis. To develop a stem cell co-culture system for HEI-OC1 cells, optimizing the temperature for the survival of both cell lines is necessary as HEI-OC1 cells would differentiate and die at the temperature at which stem cells are cultivated. Since ESCs and HEI-OC1 cells require different culture mediums and environmental conditions, pre-conditioning was required to properly sustain both cell populations for a co-culture system.

We believe the direct co-culture system of EBs was achieved because HEI-OC1 cells increased the essential factor or facilitated differentiation. Stem cells differentiate upon the influence of terminally differentiated cells via communication through gap junctions that exist between adherent cells^17,18^. Studies of HEI-OC1 cells have shown that they represent a common progenitor for hair cells and supporting cells of the OC. As such, they express cochlear hair cell markers such as Myo7a, ATOH1, prestin, BDNF, calbindin, and calmodulin, as well as supporting cell markers such as connexin 26 and FGF-R. During development of the OC, the basic helix-loop-helix transcription factor ATOH1 is considered to be the primary influence for the induction of sensory hair cells^19–21^.

Gene expression analysis showed the pattern of mid- and end- stage EBs in relation to the positive control for hair cell differentiation using exogenous growth factors. Following the loss of pluripotency, EBs showed low expression of the ectodermal marker E-Cad, and late expression of the basilar epithelial marker laminin. Otic vesicle markers PAX2 and PAX8 were similarly expressed in the same pattern as the control EBs. These outcomes may suggest that the co-culture system is not following the previously demonstrated differentiation process and is bypassing some steps due to signals produced by HEI-OC1 cells. Further studies are warranted to determine the underlying mechanism.

In a co-culture system, the stability of interactions between the different cell populations can change if the environment is altered. Here, we tested whether cell-to-cell interaction between ESCs and HEI-OC1 cells are essential to differentiation into the otic lineage or soluble substrates released by HEI-OC1 cells alone are enough to influence the change. In the non-contact co-culture, EBs failed to differentiate and broke apart. These different outcomes of monolayer and 3D culture of ESCs highlight an important role of the assisting cells. In non-contact co-culture, assisting cells interact with target cells via paracrine signaling using soluble factors. The CM from HEI-OC1 cells contain soluble factors that signal ESCs to differentiate. However, without the cell-to-cell connection between the co-cultured cells, the amount of soluble factors within the CM is dependent solely on the density of assisting cells. The higher cellular density of EBs may require a higher concentration of factors within the media, which is essential for the survival of 3D culture systems^22^. This is where direct co-culture can effectively mimic the relationships among cell types within native tissue, processes that are often inefficient when relying solely on soluble factors. Cells in contact may form cooperative relationships through gap junctions under the same culture conditions and such behavior may be halted if they are cultivated separately.

In the direct or contact co-culture system, HEI-OC1 cells assisted in the cultivation and differentiation of ESCs toward their target phenotype. The assisting cells induced the pluripotent cells to exhibit a range of desired characteristics. ESCs in the EBs successfully transformed into putative hair cells progenitors expressing otic markers PAX2 and PAX8. We were not able to confirm the mature and specific cochlear hair cell markers such as Brn3c, and Anxa4. In addition, stereocilia-like structures or expressions of FM1-43 that prove mature function of mechanoelectric transduction are yet to be found. In that sense, the source of differentiating factors which is the HEI-OC1 cell line have to be more focused. HEI-OC1 cells are developed from newly observed cell which does not fit to ordinary structures of the mouse cochlear explant which is immortalized^16^. Thus, cells were expected to be premature and able to differentiate a bit further in differentiation permissive condition and bear the characteristic of multiple cells including inner/outer hair cell, supporting cell and neural cells. Therefore, considering the facts that co-culture conditions were similar to differentiation non-permissive condition (more premature state of HE-OC1 cells) and that differentiation duration is relatively shorter than traditional differentiation process, it is possible that these differentiated cells among the co-cultured EBs are still premature and additional step is required. We believe that this co-culture technique is not superior to traditional methods since this process develops putative cells which are comparably less specific. However, because it would be difficult to adopt this immortal gene transfer technique itself to generate human HEI-OC1 cells, modifying this co-culture to generate human organoid which shared similar characteristics to the current outcome could reduce the essential factors to differentiate the transplanted cells to target cells. To achieve this future goal, we believe that factors or genes that could differentiate these putative cells to target cells should be discovered.

Research into alternative biomaterials is ongoing to efficiently use only the resulting differentiated cells^23,24^. Considering these opinions, the separation of target stem cells will lead to more effective results in subsequent experiments. In our study, it may be desirable to use stem cells separated from HEI-OC1 cells for the next stage and explants in a sensorineural hearing loss study. The stage at which transplantation of dissociated cells will lead to effective results depends on the different transplanted cell species, cell quantity, survivability, and delivery time^25^. Determining the optimal time point suitable for the purpose of the experiment will be important in increasing transplantation success^26,27^; securing reliable data will provide a basis for future human applications. Therefore, confirming the timing of transplantation of progenitor cells derived from this experiment will be important for future studies. Further studies could also be extended to using induced pluripotent stem cells (iPSCs) as a method validation for more relevant clinical translation and application in regenerative medicine.

To this extent, the maturation and functionality of SC-derived progenitor cell would be important. There are several things to be cleared before the actual usage of stem cells for transplantation. First, we have to differentiate this cell further to have complex functions such as the mechanoelectric transduction of hair cell-like cells, as confirmed by previously reported paper showing electric signal induced by the developed cells^14^ and expression of specific marker FM1-43 in our recent publication^13^. However, it is yet to replicate the fully functional specific cells that are capable of sound transduction. Second it needs to be confirmed that whether these differentiated progenitor cells are going toward cochlear or vestibular lineage and in addition whether these cells are showing both two different types of each differentiation process (cochlear inner and outer cochlear hair cells; type 1 and 2 vestibular hair cells). Although it is not yet established whether stem cell differentiation favors any of the hair cell lineage, but few evidences support the vestibular characteristics but further studies are necessary^28,29^. Lastly, connection to neural structures (synaptic connection) must be confirmed and several previous reports have confirmed the neural structure and synaptic morphologies in differentiated cells^30^.

The time point at which the stem cells should also be considered for transplantation. It is not clear whether fully matured or differentiated cells are capable to survive after cell dissociation. To confirm this issue further, study on sorting and dissociating the fully mature organoid and analyzing the survival rate and migration ability would be necessary. Prior reports that successfully sorted the progenitor cells from mammalian inner ear could be a great guide for this future experiment. If the survival rate of transplanted mature cells is poor, transplanting the precursor cells, as observed in this study, and leading them to differentiate further by applying several factors could be a useful tool. A cocktail of the HEI-OC1 cell or conditioned sera could be a candidate source for such in situ differentiation.

## Conclusion

ESCs cultivated together with HEI-OC1 using a direct co-culture system allowed for cell-to-cell contact. Both dissociated ESCs and EBs differentiated into Myo7a-positive cells. However, in the fully differentiated EBs, evidence to prove mature differentiation of inner ear hair cell was insufficient to conclude that outcome of this differentiation was still rudiment. Nevertheless, we believe an outcome of direct co-culture of EBs to generate hair cell progenitor-like cells were well achieved because HEI-OC1 cells increased an essential factor or facilitated differentiation. Since HEI-OC1 cells are developed from mouse cells, this differentiation process likely does not reflect the differentiation process in humans. With further modification and advancement of this putative technology, adopting human ESCs and establishing a co-culture system using harvested organs would be ideal. The successful development of this co-culture system should facilitate the development of a co-culture system using inner ear organs or dissociated outer and inner hair cells, which could lead to specific differentiation into cochlear-specific hair cells and possibly enable differentiation into specific outer and inner hair cells.

## Materials and methods

### ESC maintenance of embryonic body formation

In this study, we used mouse ESCs expressing enhanced green fluorescence protein (eGFP), a generous donation by Prof. Hosup Shim (Dankook University, Cheonan, Korea)^31^. The undifferentiated ESCs were cultured in feeder-free gelatin-coated plates and maintained in standard ESC medium consisting of high-glucose Dulbecco’s modified Eagle medium [DMEM; Corning, Tewksbury, MA, USA] supplemented with 15% (v/v) fetal bovine serum (FBS), 1% penicillin (ATCC, Manassas, VA, USA), 0.1 mM β-mercaptoethanol, 0.1 mM Glutamax (Gibco, Invitrogen, Carlsbad, CA, USA), 0.1 mM ES qualified non-essential amino acid (Welgene, Daegu, Korea), 0.033% CHIR99021, 0.125% PD035901 (Tocris Bioscience, Bristol, UK), and 1000 U/mL leukemia inhibitory factor (Millipore, Merck, Burlington, MA, USA). ESCs were kept at 37 °C in a humidified incubator with 5% CO_2_ at 90% humidity (Thermo Fisher Scientific, Waltham, MA, USA) and passaged every 3–4 days using TrypLE Express (Gibco; Thermo Fisher Scientific, Inc., Waltham, MA, USA).

EBs were formed using the hanging drop technique as described elsewhere^13,32^. Briefly, after dissociating ESCs with TE, drops of 30 μL with cell densities of 4 × 10^5^ cells/mL were placed onto the lid of the culture dish and cultured for 48 h under the same aforementioned conditions. The resulting EBs were collected, washed with phosphate-buffered saline (PBS), and plated for a direct or non-contact co-culture system.

### HEI-OC1 cell maintenance and preconditioning

Otic HEI-OC1 cells were cultured continuously in high glucose DMEM supplemented with 10% (v/v) FBS. The cells were maintained at a permissible condition of 33 °C in a humidified incubator under 10% CO_2_ in air and passaged every 2–3 days. HEI-OC1 cells were preconditioned and selected to be compatible for co-culture with ESCs. Cells were cultivated in ES maintenance media without LIF and streptomycin (ES-M) and incubated at 37 °C with 5% CO_2_. The cells that survived were passaged every 2–3 days using TrypLE Express. Cell proliferation was checked every passage using the 3-[4,5-dimethylthiazol-2-yl]-2.5-diphenyltetrazololium bromide (MTT) assay. A 9:1 ratio of MTT was added to each well of each passaged sample and incubated for another 4 h. The culture media was then replaced with dimethyl sulfoxide (LPS Solution, Daejeon, Korea) to dissolve the formed formazan crystals with shaking for 1 h. The optical density of the resulting purple solution was measured at a wavelength of 565 nm with a spectrophotometer (Infinite M200 Pro, Tecan, Austria). One-way ANOVA with Dunnett test (GraphPad Software ver. 9.0, Inc., La Jolla, CA, USA) was used for the analysis of the absorbance values expressed as means ± SD. P value less than 0.05 was considered to be statistically significant.

### Direct co-culture

The preconditioned HEI-OC1 cells were seeded at 3 × 10^5^ cells/mL in a FluoroDish poly-d-lysine coated glass bottom culture dish (World Precision Instruments, Sarasota, FL, USA), cultured until confluency, and deactivated using 10 µg/mL Mytomycin-C (Sigma-Aldrich, St. Louis, MO, USA) for 2 h. Meanwhile, EBs were formed using mouse GFP-positive ESCs (mGFP-ESCs) via hanging drop technique^32^. Five EBs or dissociated ESCs at 3 × 10^4^ cells/mL were co-cultured with the inactivated layer of HEI-OC1 cells in ES-M for the contact co-culture system. Half of the culture medium was changed with new medium every 2 days and kept for 14 days.

### Noncontact co-culture

We also investigated whether soluble factors alone in the culture media released by HEI-OC1 cells alone without cell-to-cell interaction could induce ESC differentiation^33,34^. The conditioned media (CM) was collected from the supernatant of pre-conditioned HEI-OC1 cells after 48 h of culture, centrifuged, and filtered. Dissociated ESCs or EBs were cultured in CM at 37 °C with 5% CO_2_ and 90% humidity. Half of the culture medium was changed with new medium every 2 days and kept for 14 days.

### Positive control for differentiated inner ear-like structures

ESCs were differentiated into inner ear-like organoids as described elsewhere^14^. Briefly, on the second day of differentiation, non-neural ectoderm was induced by adding 10 ng/mL recombinant bone morphogenetic protein 4 (BMP4) (Stemgent, Beltsville, MD, USA) and 1 μM SB431542 (Stemgent) to the cultured cells. On the third day, preplacodal ectoderm was induced by adding 25 ng/mL fibroblast growth factor (FGF)-2 (Peprotech, Rocky Hill, NJ, USA) and 1 μM LDN-193189 (Stemgent). Cells were cultured for 2 days, and the medium was replaced with maturation medium containing 1% Matrigel on day 6. Half of the medium was replaced with maturation medium without Matrigel every other day until day 14. The differentiation process is illustrated Fig. 6a.

### Immunofluorescence analysis

At the first and last days of in vitro culture, the cells or EBs were sampled for immunostaining. The samples were washed with PBS and immediately fixed with cold 4% paraformaldehyde. The samples were treated with 0.25% Triton X-100 (Sigma-Aldrich) in PBS for 10 min at room temperature (RT) for permeabilization, followed by 10% normal goat serum (NGS; Vector Laboratories, Burlington, ON, Canada) to reduce nonspecific antibody binding. Morphological changes and expression of hair cell markers for conditioned HEI-OC1 cells and co-cultures were observed through immunofluorescence staining and confocal microscopy (FW3000; Olympus, Tokyo, Japan). Primary antibodies used were Myo7a and Sox2 (1:200; Millipore, Burlington, MA, US); E-Cadherin (E-Cad, 1:250) and N-Cadherin (N-Cad, 1:100; BD Biosciences, San Jose, CA, US); Pax2, Pax8 and Epcam (1:100; Abcam, Cambridge, UK); Brn3c (1:25) and Fbxo2 (1:50; Santa Cruz Biotechnology, Dallas, TX, US); and Anxa4 (1:50; R&D Biosystems, Minneapolis, MN, US). Samples were all incubated overnight at 4 °C followed by incubation with corresponding Alexa Fluor 568 or 594 conjugated secondary antibody (1:1000; Abcam) for 1 h at RT. Cells were also stained with FITC-conjugated phalloidin (F-actin) and 4′,6-diamidino-2-phenylindole (DAPI, nuclei).

### Quantitative PCR

On the seventh and fourteenth day of contact co-culture, EBs (which were isolated from HEI-OC1 cells) were carefully isolated from the underlying layer of HEI-OC1 cells using wide-mouthed pipet tips. Conditioned HEI-OC1 cells and maintenance cultured EBs (ESC-EB) were collected for negative control, and positive control differentiated EBs (Diff. 7D, 14D) were pooled and collected. A total of 1 µg/µL RNA was isolated from five EBs or more using GeneAll Hybrid-RTM kit (GeneAll Biotechnology, Songpa-gu, Korea) followed by cDNA synthesis (Hyperscrip Master mix, GeneAll). Likewise, undifferentiated EBs and positive control differentiated EBs were also pooled and collected. A Total of 1 µg/µL RNA was isolated from cells of five EBs or more using GeneAll Hybrid-RTM kit (GeneAll Biotechnology, Songpa-gu, Korea) followed by cDNA synthesis (Hyperscrip Master mix, GeneAll). The expression of otic differentiation markers were determined by qRT-PCR analyses (SYBR Green PCR kit, Qiagen, Hilden, Germany) using AB Applied Biosystem-7500 real-time PCR system (Life Technologies, Carlsbad, CA, USA). The forward and reverse primers (Oligomer, Bioneer, Daejeon, Korea) used are listed in Table 2. One-way ANOVA with Dunnett test (GraphPad Software ver. 9.0, Inc., La Jolla, CA, USA) was used for the analysis of mRNA expressions levels indicated as means ± SD. P value less than 0.05 was considered to be statistically significant. Three independent replicate experiments were conducted.

**Table 2:** List of primers used for the qRT-PCR analysis of co-cultured embryonic bodies.

Target genes	Forward sequence	Reverse sequence
OCT4	5′-GCCTTGCAGCTCAGCCTTAA-3′	5′-AAGCCAGGAATGGAAGCAGC-3′
SOX2	5′-CACCCCTAGCCAACTTGCTG-3′	5′-CTTTGTTCTCCTCACCCGGC-3′
E-Cad	5′-TCTTAGGCACCCAGTAGGCC-3′	5′-TTCCAGGGAGACTGCTAGGC-3′
Laminin	5′-CACCCCTAGCCAACTTGCTG-3′	5′-CTTTGTTCTCCTCACCCGGC-3′
PAX2	5′-GACAGCACCAGACAAGAGGC-3′	5′-TAGCCAAAAGCCTCGGCAG-3′
PAX8	5′-CTTTGCAGTCCCCAGCTCAG-3′	5′-GCCAAGTGCTCTCCTGTGTC-3′
ATOH1	5′-TCCTATGAAGGAGGTGCGG-3′	5′-TTAGGGCCCTGTCCTCGAAG-3′
M7A	5′-CACCAAGGGAGATTGTGGCC-3′	5′-CCTTGGACACCATGACACGG-3′
GAPDH	5′-AGGTCGGTGTGAACGGATTTG-3′	5′-TGTAGACCATGTAGTTGAGGTCA-3′
